# Self-Heating Flower-like Nanoconstructs with Limited Incorporation of Yttrium in Maghemite: Effect of Chemical Composition on Heating Efficiency, Cytotoxicity and Genotoxicity

**DOI:** 10.3390/nano13050870

**Published:** 2023-02-26

**Authors:** Miloš Ognjanović, Željko Jaćimović, Milica Kosović-Perutović, Irina Besu Žižak, Tatjana Stanojković, Željko Žižak, Biljana Dojčinović, Dalibor M. Stanković, Bratislav Antić

**Affiliations:** 1 VINČA Institute of Nuclear Sciences-National Institute of the Republic of Serbia, University of Belgrade, Mike Petrovića Alasa 12-14, 11000 Belgrade, Serbia; 2 Faculty of Metallurgy and Technology, University of Montenegro, Cetinjski put bb, 81000 Podgorica, Montenegro; 3 Institute for Oncology and Radiology of Serbia, Pasterova 14, 11000 Belgrade, Serbia; 4 Institute of Chemistry, Technology and Metallurgy, National Institute of the Republic of Serbia, University of Belgrade, Njegoševa 12, 11000 Belgrade, Serbia; 5 Faculty of Chemistry, University of Belgrade, Studentski trg 12-16, 11000 Belgrade, Serbia

**Keywords:** magnetic nanoparticles, polyol synthesis, magnetic hyperthermia, cytotoxicity, comet assay

## Abstract

Partial cation substitution can significantly change the physical properties of parent compounds. By controlling the chemical composition and knowing the mutual relationship between composition and physical properties, it is possible to tailor the properties of materials to those that are superior for desired technological application. Using the polyol synthesis procedure, a series of yttrium-substituted iron oxide nanoconstructs, γ-Fe_2−*x*_Y*_x_*O_3_ (YIONs), was prepared. It was found that Y^3+^ could substitute Fe^3+^ in the crystal structures of maghemite (γ-Fe_2_O_3_) up to a limited concentration of ~1.5% (γ-Fe_1.969_Y_0.031_O_3_). Analysis of TEM micrographs showed that crystallites or particles were aggregated in flower-like structures with diameters from 53.7 ± 6.2 nm to 97.3 ± 37.0 nm, depending on yttrium concentration. To be investigated for potential applications as magnetic hyperthermia agents, YIONs were tested twice: their heating efficiency was tested and their toxicity was investigated. The Specific Absorption Rate (*SAR*) values were in the range of 32.6 W/g to 513 W/g and significantly decreased with increased yttrium concentration in the samples. Intrinsic loss power (*ILP*) for γ-Fe_2_O_3_ and γ-Fe_1.995_Y_0.005_O_3_ were ~8–9 nH·m^2^/Kg, which pointed to their excellent heating efficiency. IC_50_ values of investigated samples against cancer (HeLa) and normal (MRC-5) cells decreased with increased yttrium concentration and were higher than ~300 μg/mL. The samples of γ-Fe_2−*x*_Y*_x_*O_3_ did not show a genotoxic effect. The results of toxicity studies show that YIONs are suitable for further in vitro/in vivo studies toward to their potential medical applications, while results of heat generation point to their potential use in magnetic hyperthermia cancer treatment or use as self-heating systems for other technological applications such as catalysis.

## 1. Introduction

Nanomaterials are intensively investigated because of their potential application in various fields of technology. One of the fields of interest is medicine, where different platforms and devices are being developed for the diagnosis and treatment of various diseases [1,2,3]. When talking about the application of nanomaterials in medicine, a large group consists of magnetic nanoparticles (MNPs). Their development for medical applications led to the introduction of a new field, magnetic nanomedicine, which is primarily focused on the diagnosis and therapy of malignant diseases [4,5]. MNPs are being investigated for diagnostic applications as MRI agents, as well as for therapy, primarily as drug carriers and agents for magnetic hyperthermia (MH) [5,6]. In order to be used in this manner, MNPs need to possess desirable magnetic properties, narrow size distribution, chemical stability, low toxicity and biocompatibility.

The heat generated by MNPs when they are exposed to an external alternating magnetic field (MH) and accumulated in tumor tissue leads to a therapeutic effect when temperatures of 42–46 °C inside of tumor were reached [5]. In clinical practice, MH is used for cancer treatment by the public company MagForce Nanotechnologies AG (www.magforce.de) [7]. For MH therapy, it is necessary to deliver a sufficient amount of MNPs to the tumor tissue and they must contain a sufficiently high level of hyperthermic efficiency in order to achieve a therapeutic effect, even if smaller portions of MNPs reach tumor tissue. Hence, it is desirable to use MNPs with high efficiency in the conversion of electromagnetic field energy into heat which is expressed through the *SAR* parameter (specific heat absorption rate) which is a function of: size and size distribution of nanoparticles, magnetic properties of nanoparticles, morphology, structural defects and all forms of anisotropy [8]. One of the most investigated materials for nanomedicine applications are magnetite (Fe_3_O_4_) and maghemite (γ-Fe_2_O_3_). They have been used in various formulations approved by the USA Food and Drug Administration (FDA) [9]. Both forms of iron oxides have superior magnetic properties, can be prepared in large quantities, and are biocompatible and biodegradable, which is necessary for their biomedical application [10,11].

Some typical synthesis methods used to prepare MNPs are thermal decomposition, hydrothermal synthesis, solvothermal synthesis, etc. [12,13,14]. Using a particular method, the synthesized nanoparticles have different structural and microstructural characteristics and therefore other physicochemical properties. By changing the synthesis parameters, some of the physical properties of MNPs, which are essential for their application, can be controlled. The other way of tailoring physical properties is to change the chemical composition by doping or cationic/anionic substitution [15]. By establishing a correlation between chemical composition and (micro)structure and/or physical properties, it is possible to design materials with desired properties. The most common type of change in the chemical composition of iron oxide is the partial cation substitution of Fe^2+^/Fe^3+^ ions with ions of *3d* or *4f* elements [16]. The rare earth (*4f*) ions are larger than the hosts, and hence, they tend to create structural distortions and significantly influence the crystallite size, strain and morphology and can introduce substantial changes in anisotropy [17].

By inspecting the literature data, a significant influence of the shape of the nanoparticles on *SAR* values was reported [18]. Iron oxide nanoparticles with a flower-shaped structure obtained by the polyol-mediated route had a great heating capacity [19,20,21]. The *SAR* values of these nanoflowers were bigger by an order of magnitude than for corresponding single-domain crystallites, similar to the ones clustering in flower form [22].

A detailed analysis of the physical and chemical properties revealed that the sample obtained by the aforementioned procedure crystallizes in the structure of maghemite (γ-Fe_2_O_3_). To the best of our knowledge, the change in morphology with the incorporation of *4f* ions in the maghemite multicore structure of iron oxide prepared using the polyol method, as well as changes in microstructural and magnetic properties, maximal concentration of *4f* element for substitution Fe^3+^, the effect of heating with a change in chemical composition has not been investigated so far. One of the goals of our research was to determine the effects of the substitution of Fe^3+^ with Y^3+^ in γ-Fe_2_O_3_, particularly the changes in the heating efficacy due to the potential application of the synthesized nanoparticles in MH.

Regardless of whether one is talking about application for diagnostic or therapeutic purposes, it is necessary to accumulate a sufficient amount of IONPs into the cells. For this reason, it is crucial to investigate the biological effects of nanoparticles such as inflammation, oxidative stress, genotoxicity, etc. An inspection of the literature data indicates the importance of the surface coating of nanoparticles and their size on the mechanisms of toxicity, cellular response and the intensity of the effect [23]. The results of toxicological studies of IONPs are difficult to compare due to the lack of standardization of tests. The second goal of this research is to determine the cytotoxicity and genotoxicity of the synthesized γ-(Fe,Y)_2_O_3_.

Here, we report the synthesis of the nanoconstructs of γ-(Fe,Y)_2_O_3_ and their testing for applications in magnetic hyperthermia. High hyperthermic efficiency and low toxicity provide conditions for further functionalization of γ-(Fe,Y)_2_O_3_ and testing MH effects on cancer cell lines and tumors in small animals (mice).

## 2. Materials and Methods

### 2.1. Reagents

All reagents in this study were used as supplied without any further purification treatment. Iron(II) chloride tetrahydrate (FeCl_2_·4H_2_O), iron(III) chloride hexahydrate (FeCl_3_·6H_2_O), iron(III) nitrate nonahydrate (Fe(NO_3_)_3_·9H_2_O), yttrium chloride hexahydrate (YCl_3_·6H_2_O), diethylene glycol (DEG), N-Methyldiethanolamine (NMDEA), sodium hydroxide (NaOH), sodium chloride (HCl), nitric acid (HNO_3_), and dimethyl sulfoxide (DMSO) were all purchased from Sigma Aldrich (St. Louis, MI, USA). Trypsin-EDTA solution was purchased from Merck (Kenilworth, NJ, USA). Ultrapure Milli-Q water was used in all experiments (Millipore Co., Billerica, MA, USA).

### 2.2. Synthesis of YIONs

The yttrium-doped magnetic iron oxide nanoconstructs (YIONs) were synthesized utilizing iron chloride as the iron precursor in an aminated polyol media using an optimized polyol technique [24,25]. To design compact multicore structures (between 50–100 nm) with sufficiently large cores to enhance heating capability among the various approaches found in the literature for preparation multicore structures, we have chosen polyol-mediated route.

To prepare γ-Fe_2_O_3_, a mixture of 1.6 mmol of FeCl_2_·4H_2_O and 3.2 mmol of FeCl_3_·6H_2_O were added to 64 g of the solvent media composed of DEG and NMDEA (50/50 *w*/*w*) and vigorously stirred magnetically for 60 min. In the other beaker, 12.8 mmol of sodium hydroxide was stirred ultrasonically with 32 g of solvent mixture (50/50 *w*/*w* of DEG and NMDEA) for one hour with heating at 80 °C. After the complete dissolution of salts, the sodium hydroxide solution was carefully transferred to a solution of iron salts and stirred for one more hour. The resulting mixture was sealed in 100 mL Teflon reactor, placed in the preheated oven and allowed the reaction to progress for 12 h at 220 °C. After the synthesis, the reactor was cooled to room temperature inside the oven. Magnetic nanoconstructs were recovered by external magnet, and washed several times with a mixture of ethanol and ethyl acetate (50/50 *w*/*w*). After the synthesis, we applied acidic treatment by mechanical agitation of mixture with 10 mL of 10% nitric acid, after which 34 mmol of Fe(NO_3_)_3_·9H_2_O was added to the suspension and heated to 80 °C. Afterward, the mixture was washed once again with HNO_3_. The aim of the acidic treatment was to increase colloidal stability and to ensure the complete oxidation of the samples. At the end, the nanoconstructs were washed with deionized water, separated magnetically and redispersed in pure milliQ water. The series of YIONs was prepared by identical route, with the difference in reducing the amount of FeCl_3_·6H_2_O, and replacing it with YCl_3_·6H_2_O at various molar ratios (see Table 1).

### 2.3. Characterization Techniques

The morphology and particle size analysis of synthesized YIONs was performed on a FEI Talos F200X high-resolution transmission electron microscope (HR-TEM, Thermo Fisher Scientific, Waltham, MA, USA) at an accelerating voltage of 200 kV. Diluted water dispersions of YIONs were dropped on a carbon-coated copper grid and left to dry at room temperature for electron microscopy measurements. The morphology and size of nanoconstructs (NC) were determined by statistical analysis of TEM data, after measuring the longest YIONs diameter of about 100 NC per sample. The public domain ImageJ software package was used to examine the NC size distribution. After that, the data were fitted to a normalized logarithmic function, according to the formula:

(1) $$y=y_{0}+\frac{A}{\sqrt{2\pi }\omega x}e^{-\frac{{\left[\ln\frac{x}{xc}\right]}^{2}}{2\omega^{2}}}$$

The crystal structure of YIONs were inspected by analyzing X-ray powder-diffraction (XRPD) data measured on a high-resolution SmartLab^®^ X-ray diffractometer (Rigaku, Tokyo, Japan) equipped with CuKα radiation source. Measurements were performed under an accelerating voltage of 40 kV and 30 mA current. The dried powder of YIONs were flattened with a zero-background silicon wafer. Diffraction patterns were collected within the 10–70° 2θ range with a recording speed of 1 °/min measurement and 0.05° step size.

Measurements of magnetic hyperthermia efficacy were performed on a commercial DM 100 nB AC hyperthermia device (nanoScale Biomagnetic, Zaragoza, Spain). Calorimetric curves of colloidal water YIONs dispersions (*c* = 5 mg/mL) were recorded at various experimental conditions. The frequency used in MH measurements were in the range of 252–729 kHz, while the AC fields were from 3.98 kA/m to 19.89 kA/m. The temperature increase was monitored for 80 s using a fiber optic detector. The *SAR* and *ILP* values were calculated using the initial slope approach (*dT/dt*)_0_ by applying the corrected slope equation, Equation (2):

(2) $$SAR\left(W/g\right)=c_{NP}+\frac{\rho_{1}}{\rho_{NP}}\times c_{1}\times {\left(\frac{dT}{dt}\right)}_{max}.$$

In this equation, *C_NP_* stands for nanoconstruct-specific heat (J/(kg∙K)), *ρ_NP_* is the colloid density (Kg/m^3^), *ρ*_1_ is dispersing agent density (Kg/m^3^), while (*dT*/*dt*)*_max_* denotes heating curve temperature gradient (K/s).

### 2.4. Determination of Cell Survival

Human cervix adenocarcinoma cell line (HeLa), and the normal cell line MRC-5 (human embryonic lung fibroblasts) were grown as a monolayer in complete nutrient medium (RPMI-1640 without phenol red supplemented with 3 mM L-glutamine, 100 μg/mL streptomycin, 100 IU/mL penicillin, 10% heat-inactivated fetal calf serum (FCS), and 25 mM Hepes-pH 7.2).

Stock solutions of the investigated compounds were prepared in dimethyl sulfoxide (DMSO) and then dissolved in complete medium to achieve adequate working concentrations.

Target adherent cells HeLa (2500 cells/well), and MRC-5 (5000 cells/well) were seeded into the wells of a 96-well flat-bottom microtiter plate. Twenty-four hours later, after the cell adherence, different concentrations of investigated compounds were added to the wells, except for the controls, where only the complete medium was added. Culture medium with the corresponding concentrations of the investigated compounds, but without cells, was used as a blank. The cultures were incubated for 72 h, and the effects of the investigated compounds on cancer and normal cell survival were determined using the MTT variant of the microculture tetrazolium assay (MTA), according to Mosmann [26], with modification by Ohno and Abe [27].

Briefly, 20 μL of MTT dye solution (5 mg/mL of 3-4,5-dimethylthiazol-2-yl-2,5-diphenyltetrazolium bromide in phosphate-buffered saline) was added to each well. Samples were incubated for additional 4 h at 37 °C in a humidified atmosphere with 5% CO_2_. Afterward, 100 μL of sodium dodecyl sulfate was added to extract the insoluble formazan, which represents the product of the conversion of the MTT dye by viable cells. The number of viable cells in each well is proportional to the intensity of the absorbance (A) of light, which was measured in a microtiter plate reader at 570 nm after 24 h. To determine cell survival (S%), the A of a sample with cells grown in the presence of various concentrations of the investigated compounds was divided by the control optical density (the A of control cells grown only in nutrient medium) and multiplied by 100. The A of the blank was always subtracted from the A of the corresponding sample incubated with the target cells. All experiments were performed in triplicate.

### 2.5. Alkaline Comet Assay

Comet assay or the electrophoresis of single cells in agarose gel is a quick and sensitive method to investigate the cell DNA integrity, i.e., detection of DNA damage. MRC-5 cells were seeded in 12-well plates (15 × 10^4^ cells/well) and exposed to different concentrations of investigated compounds. Cells that were not exposed to agents served as a control. After 24 h treatment, the medium was removed and 0.005% trypsin was added to the cells and they were incubated at 37 °C for 5 min to enable their detachment from the substrate. Afterwards, a medium containing FCS was added to stop the enzymatic action of trypsin and cell density was adjusted to 1 × 10^6^ cells/mL. Cells (10 μL) were mixed with 1% low-melting point (LMP) agarose and applied onto microscopic slides pre-coated with 0.5% normal-melting point (NMP) agarose. After agarose solidification, the additional layer of 1% LMP agarose was applied on slides, followed by immersing the slides into alkaline lysis solution (1 M NaCl, 0.1 M EDTA, 10 mM Tris-HCl, PBS (Ca^2+^ and Mg^2+^ free), 1% Triton X-100, 30 mM NaOH) for 120 min at 4 °C. After a gentle wash with distilled water, the slides were immersed in cold electrophoresis buffer (300 mM NaOH, 1 mM EDTA, pH > 13) for 20 min to unfold the DNA chain at the sites of eventual labile alkali breakages. Then, electrophoresis was performed (30 min at 0.74 V/cm) in the same buffer. After the electrophoresis, slides were neutralized with 0.4 M Tris-HCl (pH 7.5) and stained with ethidium bromide (80 μg/mL). For the visualization of DNA damage, a fluorescent microscope (Axio Imager.Z1; Carl Zeiss, Jena, Germany) was used at 400× magnification.

## 3. Results and Discussion

### 3.1. Samples Preparation, Crystal Structure, Morphology and Microstructure of YIONPs

**Figure 1:**
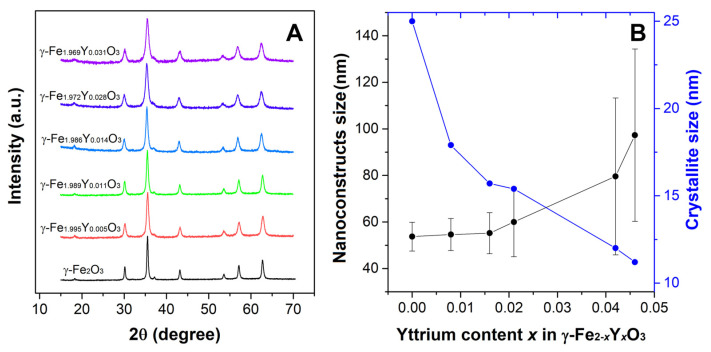
(**A**) X-ray diffraction patterns of γ-Fe_2−*x*_Y*_x_*O_3_. (**B**) Dependence of nanoflowers and crystallite size on yttrium content (*x*).

To synthesize YIONSs with a multicore structure composed of small nanocrystals arranged in a porous manner, polyol synthesis conditions were modified [24,25]. X-ray diffraction patterns of YIONs were shown in Figure 1. All reflections can be indexed in spinel structure type (space group Fd-$\bar{3}$m), indicating that the chemical composition of the investigated samples is magnetite or yttrium-substituted magnetite. However, the crystal structure of maghemite γ-Fe_2_O_3_ is close to crystal structure of Fe_3_O_4_ with vacancies presented at cation sites. Namely, magnetite tetrahedral 8*a* sites are also occupied with Fe^3+^ ions in maghemite, while octahedral sites in magnetite are occupied with both Fe^2+^ and Fe^3+^ in a ratio 1:1 differently, such as in maghemite, where 16*d* sites are occupied both with Fe^3+^ and vacancies in a 5:1 ratio. The basic structure of γ-Fe_2_O_3_ is cubic (space group P4_3_32), whereas the ordered distribution of the cation vacancies on octahedral 16*d* sites results in symmetry reduction, P4_3_32→P4_1_2_1_2 [28]. The X-ray diffraction patterns from the magnetite and maghemite matched exactly to JCPDS 19-629 and JCPDS 39-1346, respectively [29]. However, for nanopowders usually, it is not possible to distinguish the difference in diffraction patterns between magnetite and maghemite. Using the X-ray diffraction technique, some methods were proposed to analyze diffraction data and identify if the crystal structure of a sample is magnetite or maghemite. One of them is introduced by our group and it was based on two-phase Rietveld refinement [30] and the other one on the conventional peak deconvolution technique [29]. However, a detailed crystallographic analysis is not one of the objectives of the present work. For sample synthesis, we modified a protocol published by Gavilán et al. [20]. They reported a detailed analysis of the crystal structure of flower-structured nanoconstructs of iron oxide and concluded that the samples crystallized in maghemite form. Consequently, here we considered the particles to crystallize in maghemite structure and composition of γ-Fe_2−*x*_Y*_x_*O_3_.

The content of yttrium and iron in the prepared samples was determined using inductively coupled plasma with optical emission spectroscopy (ICP-OES) (Thermo Fisher Scientific, iCAP some Duo ICP, Cambridge, UK). Elements in ferrofluids, quantified after total acid digestion, were measured at Fe II 259.837 nm (Fe II) and 377.433 nm (Y II) emission wavelengths. Based on the elemental analysis, the chemical formulas of the samples were calculated (Table 1, column 2). To prepare samples, starting compounds were used according to the targeted chemical composition (Table 1, column 1). Our goal was to perform a series of *γ*-Fe_2−*x*_Y*_x_*O_3_ with different substitutions of Fe^3+^ with Y^3+^ up to the maximum content (*x* in formula unit). However, the incorporation of yttrium was less than expected with the stoichiometric ratio of starting compounds in the reaction. A maximum of 1.5% of Y^3+^ content was observed (γ-Fe_1.969_Y_0.031_O_3_). With further variation of the starting compounds’ quantities in the reaction mixture with the intention of obtaining a sample with a higher yttrium content, the crystallization and formation of the sample did not occur. It can be concluded that there is a limiting concentration of iron for substitution by yttrium in the maghemite structure, when polyol-mediated method was used. Namely, about 15–25% of the theoretical (expected) yttrium ions were incorporated (substituted Fe^3+^) into the crystal lattice. No reflections of the second phase were observed in the diffractograms of the samples, which is a clear indication that unreacted Y^3+^ was in the supernatant and was separated from the formed sample during washing steps. On the other side, the substitution of iron ions by *3d* ions in the maghemite structure is possible in a wider range of concentrations due to the similarity in ionic radii and electronic structure.

**Figure 2:**
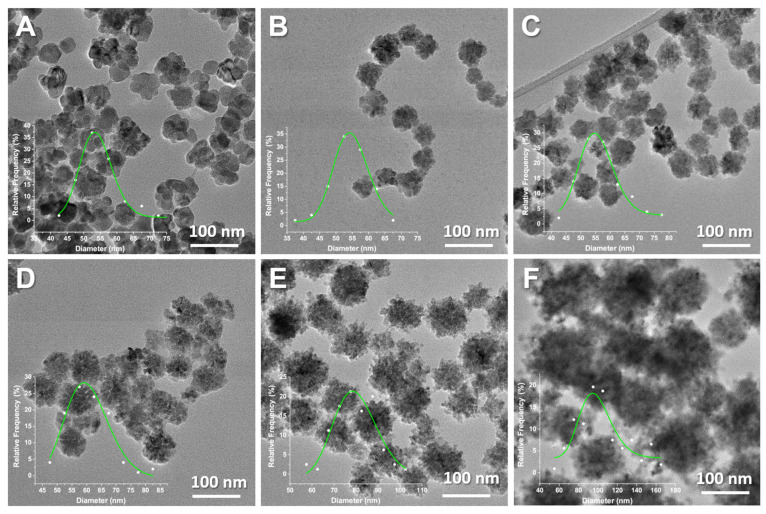
TEM images of γ-Fe_2−*x*_Y*_x_*O_3_ (**A**) *x* = 0; (**B**) *x* = 0.005; (**C**) *x* = 0.011; (**D**) *x* = 0.014; (**E**) *x* = 0.028 and (**F**) *x* = 0.031. Corresponding particles’ size distribution has been given as figure insets.

The morphology and size of nanoconstructs (NC) were determined using statistical TEM analysis (Figure 2). This analysis has been performed in order to determine the mean NC size (*D_TEM_*), standard deviation and polydispersity index (σ*_TEM_*). When it comes to NC size, σ*_TEM_* parameter is considered the absolute measurement error and is a measure of sample heterogeneity [31]. Statistical TEM analysis revealed that the agglomerated single particles form shaped flower-like structures, with the average size of YIONs ranging from (53.7 ± 6.2) nm for γ-Fe_2_O_3_ to (97.3 ± 37.0) nm for the NC at the end of the series. As can be seen from Figure 1B, there has been an evident trend of increasing nanoconstructs size with increasing yttrium share in maghemite structure. Additionally, a steep decrease in crystallite size is evident, which indicates that the individual nanocrystals that make up the nanoflowers are getting smaller (from 25 nm to 11.2 nm), as well as the fact that the polydispersity of the samples is increasing (Figure 1B and Table 1). A visible increase in nanoflowers size and an increase in inhomogeneity occurs only after *x* = 0.014. Crystallite size (*D_XRD_*) of the samples was determined by Scherrer formula using full width at half maximum (FWHM) of the most intense (311) diffraction peak.

As stated, all nanoconstructs have a flower morphology, which indicates that the incorporation of yttrium into the maghemite structure does not affect the formation of nanoflowers. However, we should keep in mind that the limit concentration of yttrium in γ-Fe_2−*x*_Y*_x_*O_3_ is low (*x* around 0.03). The reaction yield for the last sample in the series was the lowest, and the crystallite size was the smallest (Table 1). In our earlier research on the substitution of Fe^3+^ with Y^3+^ in the structure of magnetite using the precipitation method in the microwave field, a change in morphology from pseudospherical for magnetite, and the formation of nanorods in (Fe,Y)_3_O_4_ was shown, and a larger quantity of nanorods was found as the concentration of yttrium was higher [16]. These results and others found in the literature indicate that the method of iron oxide synthesis dominantly determines the shape of the particles. Additionally, the change in shape with the incorporation of yttrium ions in the iron oxide structures depends on the method of synthesis [30]. Additionally, it can be concluded that it is possible to synthesize yttrium-substituted iron oxide nanoconstructs with different concentrations of yttrium by selecting adequate synthesis method. It is useful to mention the result of the mechanochemical procedure where the sample of the chemical composition Fe_2.85_Y_0.15_O_4_ was successfully synthesized [30].

### 3.2. Heating Efficiency of YIONPs

**Figure 3:**
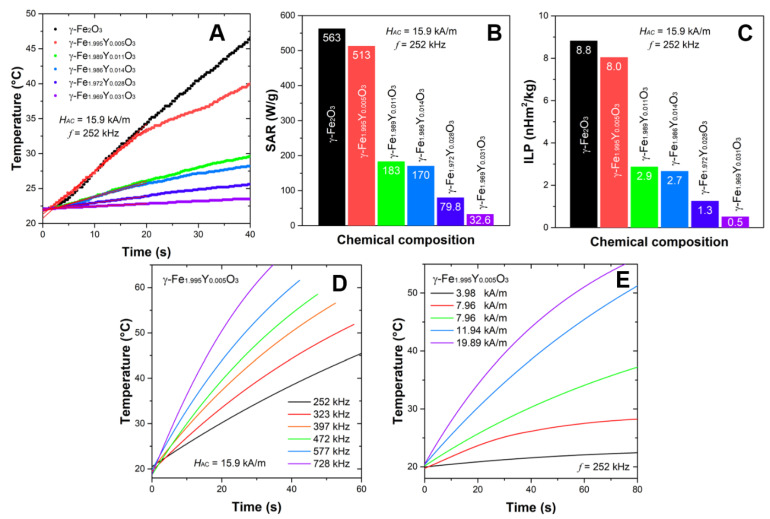
Magnetic hyperthermia of YIONs. (**A**) Calorimetric curves at *H_AC_* = 15.91 kA/m and *f* = 252 Hz; (**B**) Corresponding *SAR* values; (**C**) *ILP* of γ-Fe_2−*x*_Y*_x_*O_3_; (**D**) temperature elevation profiles for the γ-Fe_1.995_Y_0.005_O_3_ nanoflowers under different AC magnetic fields (3.98 kA/m < *H_AC_* < 19.89 kA/m) at a constant frequency, *f* = 252 Hz; (**E**) heating curves of γ-Fe_1.995_Y_0.005_O_3_ at different frequencies (252 kHz < *f* < 728 kHz) under the field of 15.9 kA/m.

The analysis of literature data shows that particles of anisometric shape show a significant increase in *SAR* value [18]. When comparing the heating efficiency of MNPs with different shapes using the reported *SAR* values, a significant influence of shape was found: *SAR*_Nanocubes_ > *SAR*_Nanoflowers_ > *SAR*_Nanooctahedra_ > *SAR*_Nanorods_ [18]. The heating curves of the studied flower-structured YIONs displayed in Figure 3A show that in only about 30 s temperature above that required for the treatment of malignant tumors with magnetic hyperthermia is reached, which indicates that γ-(Y,Fe)_2_O_3_ can be used as heating agents in magnetic hyperthermia applications. The *SAR* values are of ~500 W/g (for *H_AC_* = 15.91 kA/m and *f* = 252 KHz) for samples at the beginning of the series, with a tendency to rapidly decrease with increasing Y^3+^ concentration, Figure 3B. Substitution of magnetic Fe^3+^ with diamagnetic Y^3+^ in γ-Fe_2_O_3_ is expected to decrease the saturation magnetization of mixed samples in comparison with the parent compound and hence decrease in *SAR* values. However, *SAR* also depends on other parameters such as the distribution of yttrium ions in the crystal structure of maghemite, the influence of substitution on the microstructural parameters (size and microstrain), the distribution of nanoconstructs by size, hydrodynamic diameter, colloidal stability and anisotropy of nanoflowers. It was found that the size of the nanoconstructs depends on the yttrium concentration, Table 1. This parameter affects the Brownian contribution to heat losses in MH [32].

Flower-structured iron oxide with maghemite and magnetite structure achieved very high *SAR* values (500–2000 W/g) in MH, as was reported in the literature [18]. Usually, measurements have been performed under different *H* and *f*, so the one needs to be careful when comparing reported data. The other MH parameter that offers a better insight into the heating ability of MNPs is intrinsic loss power (*ILP*), which is defined by the equation *ILP* = *SAR*/*H^2^f*. The *ILP* values for the samples in this study are in the range 0.5–8.8 nH·m^2^/Kg, Figure 3A. Samples *γ*-Fe_2_O_3_ and γ-Fe_1.995_Y_0.005_O_3_ with of ~8–9 nH·m^2^/Kg are excellent candidates for MH applications (in vitro and in vivo). When comparing the numerous reported values of *SAR* and *ILP*, the fact that the measurements are carried on different devices without prior calibration should be taken into account. It is useful to mention that as part of the COST action [RADIOMAG TD1402], magnetic hyperthermia measurements were performed on the two identical stable nanoparticle systems in an interlaboratory study (across *N* = 21 European sites). The conclusion of this COST action was a lack of harmonization and standard deviations of results from ±30% to ±40% [33]. It is worth mentioning the work of Bender et al. [34]. They performed a critical study about the interdependence between the microstructure, magnetism, relaxation dynamics and heating efficiency of nanoflowers. They showed that, except for Néel and Brownian relaxations, the relaxation of the disordered spins within the nanoflowers substantially influences high *ILP* values. Previously, in the works of Dutz et al. and Lartigue et al., the excellent heating ability of nanoflowers was explained by exchange coupling between the cores (crystallites or particles), which are in direct contact with each other within a multicore flower-like structure, leaning them to a superferromagnetic magnetization state [24,35]. The enhancement of nanoflower magnetization leads to higher magnetic losses in magnetic hyperthermia compared to small cores [36].

When speaking about MH for the treatment of malignant tumors, the hyperthermic efficiency of nanoparticles should be observed in the medically safe range of frequencies and field strengths. The limits of the *H × f* factor for cancer treatment by MH in a clinic is less than 4.85 × 10^8^ (A m^−1^ s^−1^) according to the Atkinson–Brezovich criteria [37]. Figure 3D,E shows the results of temperature increase with time for the selected sample γ-Fe_1.995_Y_0.005_O_3_ in different AC fields and various frequencies. It was shown that high temperatures can be reached in the fluid within few seconds. This result indicates that the YIONs as a hyperthermic agent can be used for more specific applications such as catalysis [38].

### 3.3. Toxicity of YIONs

**Figure 4:**
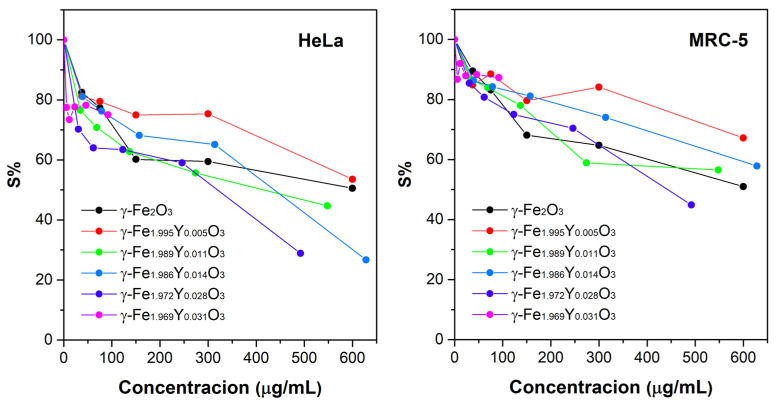
Survival of malignant HeLa and normal control MRC-5 cells, as determined by MTT test, after 72 h of continuous action of applied concentrations of YIONs. It is given as a function of different concentrations of investigated compounds.

Investigated YIONs with *x* = 0.011, *x* = 0.014 and *x* = 0.028 exerted weakly moderate antiproliferative action toward target tumor cell lines (Figure 4 and Table 2). All other constructs were inactive over the range of concentrations applied. All nanoconstructs were inactive against normal cells, except YIONs with *x* = 0.028, which was the most active against tumor cells as well, and had somewhat lower activity towards normal than tumor cells (IC_50_ = 432 ± 12 µg/mL against MRC-5 cells, and IC_50_ = 324 ± 4 µg/mL against HeLa cells). The synthesis yield of *x* = 0.031 was much lower than others; therefore, the highest applied concentration for MTT assay was 90 µg/mL. Up to this concentration, these nanoconstructs did not express any cytotoxic behavior. The cytotoxic effect is explained by the large surface area of nanoparticles with a disordered crystal structure, broken chemical bonds and a large number of ions, Fe^2+^ and Fe^3+^. These ions as electron donors or acceptors accelerate the formation of free radicals [39]. A greater accumulation of nanoparticles in cells leads to a higher amount of iron ions and excessive formation of free radicals, and therefore to damage cell compartments, oxidize lipids, proteins and DNA [39]. The good colloidal stability achieved by the polyol technique does not require additional coating in order to increase colloidal stability and prevent agglomeration [25,40]. On the other side, the cytotoxicity analysis indicates that it is not necessary to coat the nanoconstructs to reduce their toxicity, and they can be used as prepared in further in vivo studies. High hyperthermic efficiency point to that in order to achieve a therapeutic effect with magnetic hyperthermia, it is not necessary to administer a high concentration of nanoconstructs, thus preventing their possible toxic effect on healthy cells.

### 3.4. Comet Assay for Genotoxicity Evaluation

**Figure 5:**
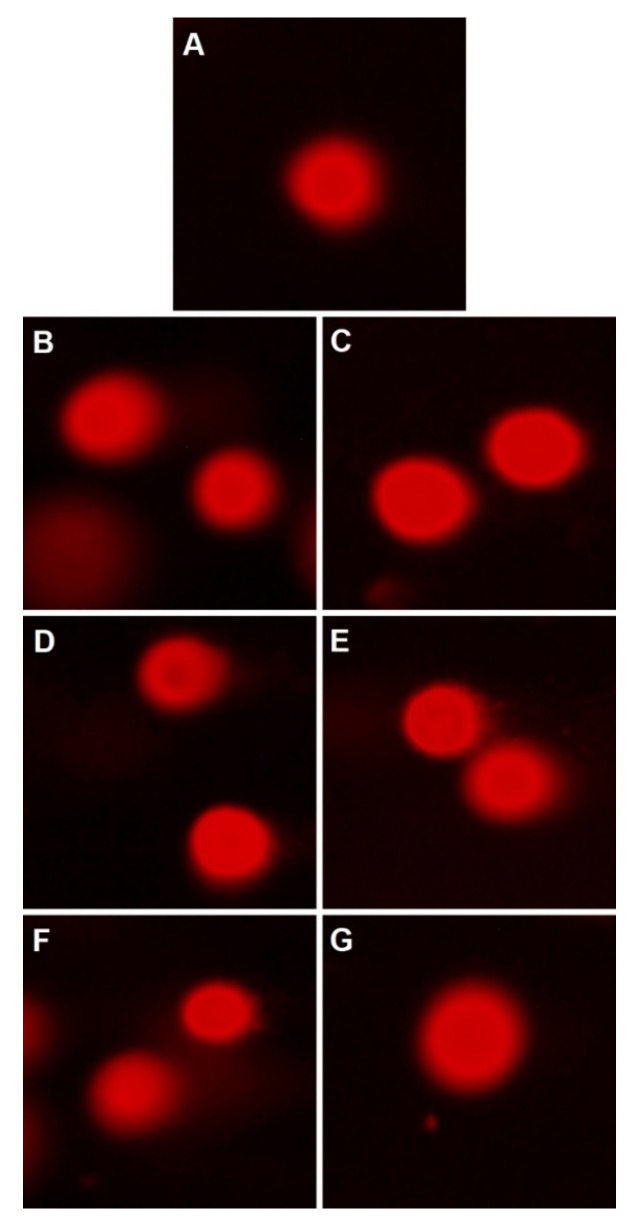
Comet assay of γ-Fe_2−*x*_Y*_x_*O_3_ performed on MRC-5 cell line. (**A**) Control; (**B**) *x* = 0; (**C**) *x* = 0.005; (**D**) *x* = 0.011; (**E**) *x* = 0.014; (**F**) *x* = 0.028 and (**G**) *x* = 0.031.

Comet assay performed after the treatment of normal MRC-5 cells for 24 h with all investigated nanoconstructs at concentrations that correspond to IC_50_ obtained in MTT assay revealed that all investigated samples did not exhibit genotoxic effects (Figure 5). That being said, from the cytotoxic and genotoxic analysis, the prepared YIONs showed the potential to be further tested in vitro and in vivo as candidates for medical applications.

## 4. Conclusions

Using the polyol-mediated method, it was shown that it is possible to substitute iron ions with yttrium ions up to about 1.5% in γ-Fe_2_O_3_. The incorporation of yttrium into the crystal lattice of maghemite had an impact on the hyperthermic efficiency of studied nanoconstructs. *SAR* or *ILP* parameters decrease significantly in mixed samples of γ-Fe_2−*x*_Y*_x_*O_3_ (*x* ˃ 0.005). The *ILP* values of 8.8 and 8.0 nH·m^2^/Kg for γ-Fe_2_O_3_ and γ-Fe_1.995_Y_0.005_O_3_, respectively, are significantly high, indicating that these YIONs are promising for in vivo applications as agents in magnetic hyperthermia. Additionally, it was shown that the examined YIONs do not exhibit toxic activity towards normal cells, which is significant for medical applications. Keeping in mind that ^90^Y is a therapeutic radionuclide that is used in our group for the radiolabeling of nanoparticles for the purpose of designing potentially new radiopharmaceuticals, the incorporation of ^90^Y into the maghemite structure and the formation of γ-Fe_2−*x*_^90^Y*_x_*O_3_ is expected to be achieved by using the polyol method, which could be a potential bi-modal agent for simultaneous radionuclide and MH cancer therapy. Here, synthesized magnetic multi-core nanoconstructs show a dependence of properties on the chemical composition. Partial cation substitution can be used to fine-tune, first of all, the microstructural and magnetic properties for the purpose of their specific applications, in this case a biomedical one.

## Figures and Tables

**Table 1:** Chemical composition of YIONs according stoichiometric ratio of starting compound (targeted) and actual composition determined by ICP-OES. Ferrofluid concentration *c*, crystallite size *D_XRD_*, average nanoconstructs size *D_TEM_* and polydispersity index σ*_TEM_* are listed.

Chemical Composition (Targeted)	Chemical Composition (ICP-OES)	*c* (mg/mL)	*D_XRD_* (nm)	*D_TEM_* (nm)	σ*_TEM_* (%)
γ-Fe_2_O_3_	γ-Fe_2_O_3_	16.0	25.0	53.7 ± 6.2	8.99
γ-Fe_1.966_Y_0.034_O_3_	γ-Fe_1.995_Y_0.005_O_3_	15.0	17.9	54.6 ± 6.9	9.74
γ-Fe_1.931_Y_0.069_O_3_	γ-Fe_1.989_Y_0.011_O_3_	13.7	15.7	55.2 ± 8.8	10.37
γ-Fe_1.949_Y_0.051_O_3_	γ-Fe_1.986_Y_0.014_O_3_	15.7	15.4	60.0 ± 14.9	12.45
γ-Fe_1.895_Y_0.105_O_3_	γ-Fe_1.972_Y_0.028_O_3_	12.3	12.0	79.6 ± 33.7	12.51
γ-Fe_1.778_Y_0.222_O_3_	γ-Fe_1.969_Y_0.031_O_3_	2.3	11.2	97.3 ± 37.0	16.91

**Table 2:** IC_50_ values of investigated nanoconstructs against cancer and normal cells.

Chemical Composition	IC_50_ (μg/mL) Av ± SD *	
	HeLa	MRC-5
γ-Fe_2_O_3_	>600	>600
γ-Fe_1.995_Y_0.005_O_3_	>600	>600
γ-Fe_1.989_Y_0.011_O_3_	429 ± 13	>600
γ-Fe_1.986_Y_0.014_O_3_	412 ± 25	>600
γ-Fe_1.972_Y_0.028_O_3_	324 ± 3	432 ± 12
γ-Fe_1.969_Y_0.031_O_3_	>90	>90

* From tree different experiments.

## Data Availability

The study did not report any data.
